# Identification of LPS-Activated Endothelial Subpopulations With Distinct Inflammatory Phenotypes and Regulatory Signaling Mechanisms

**DOI:** 10.3389/fimmu.2019.01169

**Published:** 2019-05-24

**Authors:** Erna-Zulaikha Dayang, Josée Plantinga, Bram ter Ellen, Matijs van Meurs, Grietje Molema, Jill Moser

**Affiliations:** ^1^Medical Biology Section, Department of Pathology and Medical Biology, University Medical Center Groningen, University of Groningen, Groningen, Netherlands; ^2^Department of Critical Care, University Medical Center Groningen, University of Groningen, Groningen, Netherlands

**Keywords:** endothelial cells, HUVEC, lipopolysaccharide (LPS), adhesion molecules, endothelial heterogeneity, intracellular signaling

## Abstract

Sepsis is a life-threatening condition caused by a dysregulated host response to infection. Endothelial cells (EC) are actively involved in sepsis-associated (micro)vascular disturbances and subsequent organ dysfunction. Lipopolysaccharide (LPS), a Gram-negative bacterial product, can activate EC leading to the expression of pro-inflammatory molecules. This process is molecularly regulated by specific receptors and distinct, yet poorly understood intracellular signaling pathways. LPS-induced expression of endothelial adhesion molecules E-selectin and VCAM-1 in mice was previously shown to be organ- and microvascular-specific. Here we report that also within renal microvascular beds the endothelium expresses different extents of E-selectin and VCAM-1. This heterogeneity was recapitulated *in vitro* in LPS-activated human umbilical vein EC (HUVEC). Within 2 h after LPS exposure, four distinct HUVEC subpopulations were visible by flow cytometric analysis detecting E-selectin and VCAM-1 protein. These encompassed E-selectin^−^/VCAM-1^−^ (–/–), E-selectin^+^/VCAM-1^−^ (E-sel+), E-selectin^+^/VCAM-1^+^ (+/+), and E-selectin^−^/VCAM-1^+^ (VCAM-1+) subpopulations. The formation of subpopulations was a common response of endothelial cells to LPS challenge. Using fluorescence-activated cell sorting (FACS) we demonstrated that the +/+ subpopulation also expressed the highest levels of inflammatory cytokines and chemokines. The differences in responsiveness of EC subpopulations could not be explained by differential expression of LPS receptors TLR4 and RIG-I. Functional studies, however, demonstrated that the formation of the E-sel+ subpopulation was mainly TLR4-mediated, while the formation of the +/+ subpopulation was mediated by both TLR4 and RIG-I. Pharmacological blockade of NF-κB and p38 MAPK furthermore revealed a prominent role of their signaling cascades in E-sel+ and +/+ subpopulation formation. In contrast, the VCAM-1+ subpopulation was not controlled by any of these signaling pathways. Noteworthy is the existence of a “quiescent” subpopulation that was devoid of the two adhesion molecules and did not express cytokines or chemokines despite LPS exposure. Summarizing, our findings suggest that LPS activates different signaling mechanisms in EC that drive heterogeneous expression of EC inflammatory molecules. Further characterization of the signaling pathways involved will enhance our understanding of endothelial heterogeneous responses to sepsis related stimuli and enable the future design of effective therapeutic strategies to interfere in these processes to counteract sepsis-associated organ dysfunction.

## Introduction

Sepsis is defined as a life-threatening organ dysfunction caused by a dysregulated host response to infection (1). Despite diagnostic advances in early recognition, sepsis often escalates to multiple organ dysfunction syndrome (MODS), leading to poor outcome. Sepsis affects around 30 million people yearly worldwide, resulting in 6 million deaths (2). Although several mechanisms have been suggested to contribute to the pathophysiology of sepsis, it is still incompletely understood, thereby hindering the development of successful treatment options for patients with sepsis and sepsis-induced multiple organ dysfunction syndrome.

Endothelial cells (EC) line all blood vessels and are one of the first cells to respond to microbial product such as Lipopolysaccharide (LPS) (3). During sepsis, EC respond to LPS via the activation of pattern recognition receptors (PRRs), producing pro-inflammatory cytokines, chemokines, and adhesion molecules (4). Adhesion molecules such as E-selectin, VCAM-1, and ICAM-1 facilitate endothelial cell—leukocyte interactions, resulting in leukocyte recruitment into inflamed tissues leading to subsequent impairment of organ function (5). Therapeutic strategies designed to attenuate EC production of these inflammation associated molecules to prevent leukocyte adhesion and transmigration, may lead to diminished organ failure in patients with sepsis.

Previous studies from our laboratory have shown that in mice, LPS or TNF-α exposure results in organ and microvascular compartment specific expression of adhesion molecules (6–8). Within the kidney, at the microvascular compartment level, TNF-α induced expression of E-selectin is abundant in glomeruli, but low in the arterioles. In contrast, VCAM-1 is highly expressed in the arterioles, while expressed to a lesser extent in the glomeruli (6, 7). Our knowledge on inter- and intra-organ endothelial heterogeneity has expanded in recent years, yet how these heterogeneous responses are molecularly controlled following LPS challenge still remains elusive.

Toll-Like Receptor 4 (TLR4) (9) and the more recently identified retinoic acid inducible gene-I (RIG-I) (10) are pattern recognition receptors which have been shown to drive endothelial responses to LPS. The recognition of LPS via these receptors results in the induction of endothelial adhesion molecules, cytokines, and chemokines via several downstream signaling pathways, that include NF-κB (10–12), and p38 MAPK (12, 13). Although both NF-κB and p38 MAPK have been implicated in controlling LPS-mediated inflammatory responses, it is unknown whether one, the other, both, or other signaling pathways regulate individual EC responses at a cellular level. Likewise, and of interest in relation to the microvascular compartment specific responses of the endothelium to LPS challenge *in vivo*, whether all EC are equipped with similar downstream signaling machinery remains to be elucidated.

Here, we investigate the molecular control of the early stages of EC activation by LPS in relation to the previously reported heterogeneous EC responses found *in vivo*. We focused on LPS induced expression patterns of E-selectin and VCAM-1 within mouse renal microvascular compartments *in vivo* and their patterns in HUVEC *in vitro* using immunofluorescent staining and flow cytometry, respectively. Based on the outcome we next investigated *in vitro* whether different stages of cell division could explain the observed heterogeneous subpopulations formed, and whether these subpopulations behaved differently because of differential expression of LPS signaling machinery components. Additionally, we used pharmacological tools to examine the activity of different kinase signaling pathways to explain heterogeneous subpopulation formation in HUVEC upon LPS exposure.

## Materials and Methods

### Mice

Male C57BL/6 mice purchased from Envigo (Horst, The Netherlands) and housed in a specific pathogen-free facility, maintained on chow and water *ad libitum*, and housed in temperature-controlled chambers (24°C) with a 12 h light/dark cycle. Mice were challenged with intraperitoneal (i.p) injection of 1 mg/kg LPS [*E. coli*, serotype O26:B6 (15,000 EU/g), Sigma-Aldrich, St. Louis, MO, USA] as described elsewhere (14). Control mice were i.p administered with the same volume of 0.9% NaCl. Mice were terminated under isoflurane anesthesia 4 h after LPS challenge. Blood was subsequently drawn via cardiac puncture and the organs harvested, snap-frozen on liquid nitrogen and stored at −80°C until further analysis. All experiments were performed in compliance with the animal ethics committee of the University of Groningen.

### Endothelial Cell Culture and Stimulation

Human umbilical vein endothelial cells (HUVEC) and human lung microvascular endothelial cells (HMVEC-L) were purchased from Lonza (Lonza, Breda, the Netherlands) and cultured in EBM-2 medium supplemented with EGM-2 MV Single Quot Kit Supplements and Growth factors (Lonza) at 37°C with 5% CO_2_/95% air conditions until passage 5 at the UMCG Endothelial Cell Facility. Additionally, HUVEC were isolated from umbilical cords and cultured on 1% gelatin coated plates with RPMI 1640 medium (Lonza) supplemented with 20% heat inactivated FCS, 2 mM L-glutamine, 5 U/ml heparin, 50 μg/ml endothelial growth factor, and antibiotics (100IE/penicillin and 50 μg/ml streptomycin). HUVEC and HMVEC-L were seeded in 6 or 12 well plates. HUVEC were 70% confluent on the day of siRNA transfection while they were confluent when treated with LPS (E. coli, serotype O26:B6; E. coli, serotype O111:B4, Sigma Aldrich, St. Louis, MO) at 1 μg/mL for 4 h unless indicated otherwise.

### Cryosection Immunofluorescence Staining

Five-micrometer cryosections from snap-frozen mouse kidneys were fixed in acetone for 10 min. The sections were blocked with 0.00125% H_2_0_2_ (Merck, Darmstadt, Germany) in demineralized H_2_0 for 10 min, and subsequently blocked with 3% bovine serum albumin (BSA) in phosphate buffered saline (PBS) (w/v) for 30 min. Kidney sections were incubated at room temperature (RT) for 1 h with primary rat-anti-mouse E-selectin antibody (10 μg/mL, clone MES-1, kindly provided by Dr. D Brown, UCB Celltech, Brussels, Belgium) diluted in 5% fetal calf serum (FCS) (Sigma, St. Louis, Missouri, USA) in PBS. After washing, sections were incubated with rabbit anti-rat IgG (Vector Laboratories Inc, Burlingame, CA, USA) in 1% normal mouse serum (NMS) (Sanquin, Amsterdam, NL)/5% FCS in PBS for 45 min. The sections were then washed and incubated with anti-rabbit HRP polymer (Dako, Carpentaria, CA, USA) for 30 min. After washing, the sections were exposed to Alexa Fluor®555 Tyramide reagent prepared according to the manufacturer's instructions (#B40955, Thermo Fisher Scientific, Carlsbad, CA, USA). For the sequential second primary antibody incubation, the sections were washed and blocked again with 3% (w/v) BSA in PBS for 30 min. The second primary antibody, rat anti-mouse VCAM-1 (hybridoma supernatant of clone M/K-1.9, ATCC, Manassas VA, USA) was added to the sections for 1 h at RT. After washing, the sections were incubated with 20 μg/mL of goat anti-rat antibody conjugated with Alexa Fluor®488 (Thermo Fisher Scientific) / DAPI (1.5 μg/mL; Thermo Fisher Scientific) in 5% FCS in PBS for 45 min, washed, and mounted with Aqua/polymount (Polysciences Inc, Warrington, PA, USA). Isotype controls included rat IgG2a and rat IgG1 (10 μg/mL; both from Antigenix America Inc., Huntington Station, NY, USA). Images were taken using a Leica SP8 confocal laser scanning microscope (Leica Microsystems Ltd., Germany) with objective and numerical apertures of 40X and 1.3, respectively. Image cubes were recorded with appropriate filters using Leica application suite X software. Peak emission for Alexa Fluor 488 (VCAM-1) was at 550 nm and for Alexa Fluor 555 (E-selectin) at 610 nm. All images were captured with equal exposure times and then analyzed using Imaris image analysis software (Bitplane AG, Zurich, Switzerland).

For CD31/E-selectin respectively CD31/VCAM-1 double staining, the kidney cryosections were blocked with peroxidase and 3% BSA as described above, incubated with rat-anti mouse CD31 antibody (0.15 μg/mL, # 550274 BD Pharmingen, Franklin Lakes, NJ, USA) diluted in 5% FCS in PBS for 1 h, and subsequently stained with rat anti-mouse antibody E-selectin, or rat anti-mouse VCAM-1 according to the above-described protocols. The images were taken with a Leica DM4000B fluorescence microscope equipped with a Leica DFC345FX digital camera (Leica Microsystems Ltd., Germany) and Leica LAS V4.5 Image Software at 100X magnification with equal exposure times.

### Flow Cytometry

We employed flow cytometry to determine the expression of endothelial adhesion molecules. HUVEC or HMVEC-L were briefly washed with sterile PBS, trypsinized with trypsin-EDTA (0.025%) and washed with ice-cold wash buffer (5% FCS in PBS). Cells were then transferred to FACS tubes, washed and resuspended in 3% v/v PE-conjugated mouse anti-human E-selectin (Cat. No #322606, BioLegend, San Diego, CA, USA) and APC-conjugated mouse anti-human VCAM-1 (Cat. No #305810, BioLegend) antibodies in wash buffer for 30 min on ice in the dark. For intracellular staining, HUVEC were fixed with Fixation Reagent A (#GAS004, Thermo Fisher Scientific) for 15 min, washed, and subsequently incubated with perm buffer Reagent B (Thermo Fisher Scientific) and 3% (v/v) Brilliant Violet 421-conjugated anti-Ki67 (#330505, Biolegend, San Diego, CA, USA) antibody in wash buffer for 30 min, on ice in the dark. The cells were then washed and resuspended in wash buffer and analyzed using a MACSQuant Analyzer 10 flow cytometer (Miltenyi Biotech, San Diego, CA, USA). Data analysis was performed using Kaluza Flow analysis software (v.2.1) (Beckman Coulter, Brea, CA, USA) or FlowJo software (v.10) (Ashland, OR, USA). Isotype control antibodies mouse IgG1κ-BV421 (Cat No #400157, Biolegend), IgG2aκ-PE (Cat No #2-4724-42, eBioscience, San Diego, CA, USA), and mouse IgG1 (Cat No # IC002A, R&D System, Minneapolis, MN, USA) were used to correct for signals from non-specific binding.

### Fluorescence-Activated Cell Sorting (FACS)

HUVEC treated with LPS for 4 h were trypsinized, washed and subsequently incubated with PE-conjugated mouse anti-human E-selectin and APC-conjugated mouse anti-human VCAM-1 antibodies as described above. After the staining procedure, the cells were sorted into 4 subpopulations based on the staining pattern of E-selectin and VCAM-1 using a MoFlo Astrios FACS machine (Beckman Coulter, Brea, CA, USA): “quiescent” E-sel^−^/VCAM-1^−^ (-/-), E-sel^+^/VCAM^−^1- (E-sel+), E-sel^+^/VCAM-1^+^ (+/+), and E-sel^−^/VCAM-1^+^ (VCAM-1+) subpopulations (see **Figure 4A**). After sorting, the purity of each collected population of cells was verified by flow cytometry before subsequent analyses were performed (data not shown).

Trypsinization *per se* did not alter detection of membrane expression of the adhesion molecules in flow cytometry, as detachment of endothelial cells with versene, a gentle non-enzymatic cell dissociation reagent, showed similar patterns of endothelial surface protein expression (data not shown).

### Gene Expression Analysis by RT-qPCR

Total RNA from the cells was isolated using the RNeasy® mini kit (Qiagen, Venlo, The Netherlands) according to the manufacturer's protocols. RNA concentration (OD 260) and purity (OD260/OD280) was determined using a NanoDrop® ND-1000 UV-Vis spectrophotometer (NanoDrop Technologies, Rockland, ME, USA). Samples with an OD260/OD280 ratio of ≥1.8 were included in the analysis. cDNA synthesis was performed as previously described (15). Quantitative (q)PCR was performed in a ViiA 7 PCR System (Applied Biosystems Nieuwerkerk aan den IJssel, The Netherlands) using the following assay-on-Demand primers (Applied Biosystems,): GAPDH (assay ID Hs99999905_m1), E-selectin (assay ID Hs00174057_m1), VCAM-1 (assay ID Hs00365486_m1), TLR4 (assay ID Hs00152939_m1), RIG-I (assay ID Hs00204833_m1), MAVS (assay ID Hs00920075), IL-6 (assay ID Hs00174131_m1), IL-8 (assay ID Hs00174103_m1), MCP-1 (assay ID Hs00234140_m1), CXCL10 (assay ID Hs01124251_g1), and CXCL6 (assay ID Hs00605742_g1). Duplicate analyses were performed for each sample and the obtained threshold cycle values (C_T_) averaged. All genes were normalized to the expression of housekeeping gene GAPDH, yielding the ΔC_T_ value. The relative mRNA level was calculated by 2^−Δ*CT*^.

### siRNA–Mediated Gene Silencing

TLR4, RIG-I, and MAVS were knocked down in HUVEC using FlexiTube siRNA (Qiagen). AllStars negative control siRNA (Qiagen) was used as a negative control for all RNA interference experiments. Transient transfection was performed using Lipofectamine 2000 (Life Technologies, Carlsbad, CA, USA) according to the manufacturer's instructions. Knockdown of these genes did not diminish endothelial cell viability as described previously (10).

### Pharmacological Inhibition of Signaling Pathways

BAY11-7082 (BAY) (Alexis Biochemicals, San Diego, CA, USA) was dissolved in DMSO as a 20mM stock solution, while LY2228820 (LY) (MedChemExpress, Monmouth Junction, NJ, USA) was dissolved in DMSO as a 10 mM stock solution according to manufacturers' instructions. Stocks were stored at −80°C until needed. Prior to the experiment, BAY and LY stock solutions were diluted in HUVEC culture medium. HUVEC were pre-treated with 10 μM of BAY 30 min before, and/or 10 μM of LY 1 h before LPS stimulation. The viability and morphology of HUVEC were assessed microscopically before and after pre-treatment with either BAY or LY, and they were found to be of normal cobble-stone morphology throughout the experiments.

### Statistical Analysis

Statistical analysis of results was performed by two-tailed unpaired Student *t*-test, or one-way ANOVA followed by Bonferroni *post hoc* analysis to compare multiple replicate means. All statistical data were analyzed using GraphPad Prism software v.7 (GraphPad Prism Software Inc., San Diego, CA, USA). Differences were considered significant when *p* < 0.05.

## Results

### Distinct Expression Patterns of E-Selectin and VCAM-1 Are Found Within Kidney Microvascular Compartments of LPS-Treated Mice

**Figure 1:**
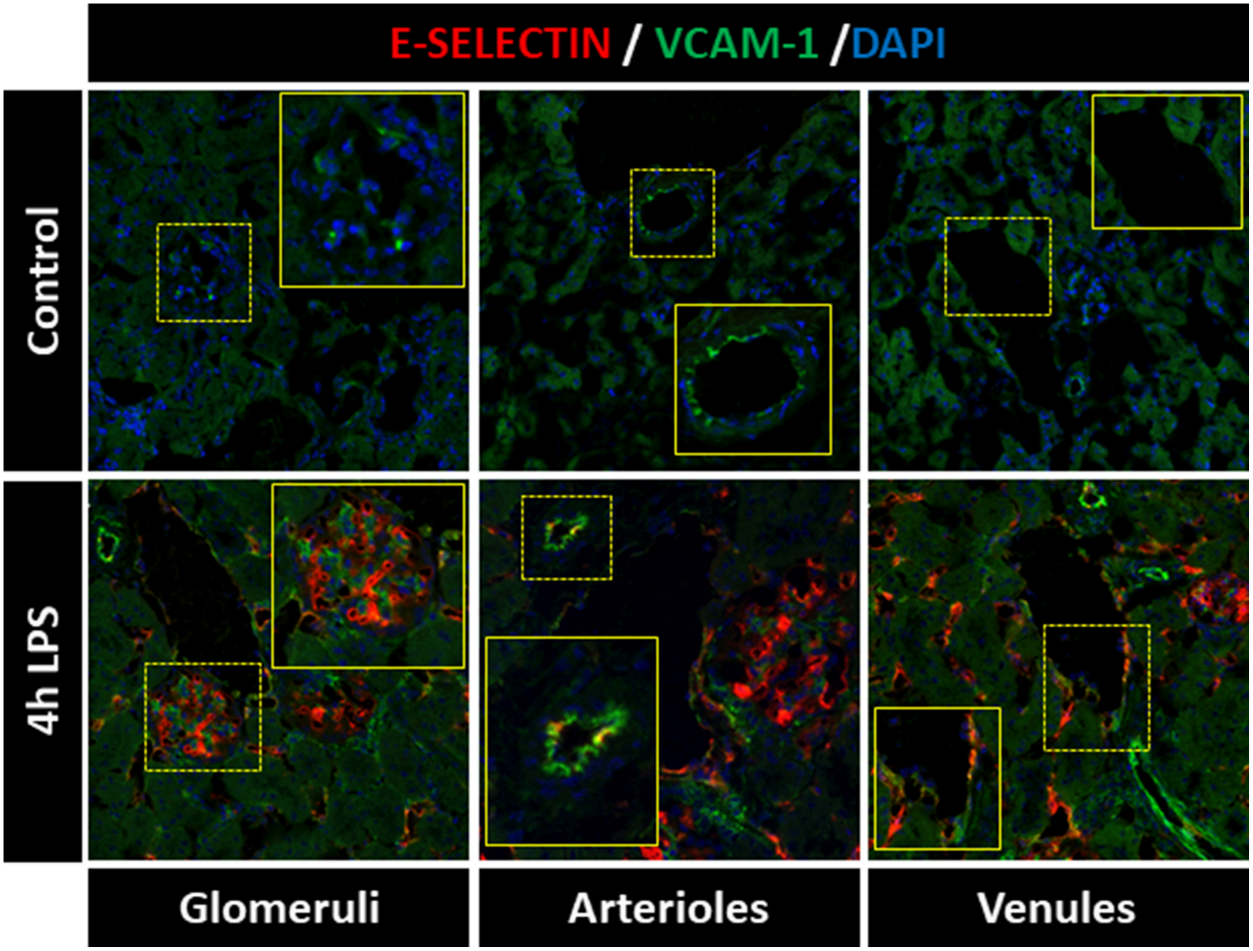
Distinct expression patterns of E-selectin and VCAM-1 are found within kidney microvascular compartments of LPS-treated mice. E-selectin and VCAM-1 expression varied between and within the renal microvascular segments of LPS-treated mice. The images show immunofluorescence staining of E-selectin (red), VCAM-1 (green), and DAPI nuclear staining (blue) in the kidney of control mice (vehicle treated) and mice challenged with LPS (1 mg/kg, i.p.) and sacrificed 4 h later. All images were captured with equal exposure times. Original magnification x400.

Heterogeneous expression of E-selectin and VCAM-1 between different renal microvascular compartments has been previously reported. Intravascular heterogeneity in expression of endothelial adhesion molecules within specific renal microvascular beds, on the other hand, is not extensively described. To investigate the expression patterns of E-selectin and VCAM-1 in the different kidney microvascular segments, we performed immunofluorescent double staining to detect these two adhesion molecules at the same time. In control kidney, E-selectin protein was absent from all microvascular compartments, while arterioles expressed basal VCAM-1 protein Figure 1, confirming previous data from our laboratory (7, 16). Following LPS challenge, E-selectin and VCAM-1 became visible in all kidney microvascular segments. However, E-selectin was mainly expressed in the glomeruli, whereas the arterioles predominantly expressed VCAM-1, with scattered co-expression of E-selectin. The peritubular capillaries and post-capillary venules mainly co-expressed E-selectin and VCAM-1, while occasional single E-selectin or VCAM-1 positive cells were found Figure 1. Double immunostaining of CD31, a pan-endothelial marker and E-selectin respectively VCAM-1 confirmed their expression by endothelial cells in the microvascular segments (Supplementary Figure 1). These data demonstrate that EC in different microvascular compartments, and also within the same microvascular compartment, exert a heterogeneous phenotype which can be identified by different patterns of E-selectin and VCAM-1 protein expression following LPS challenge.

### LPS Stimulation of Endothelial Cells *in vitro* Uncover Cell Subpopulations Based on E-Selectin and VCAM-1 Expression

**Figure 2:**
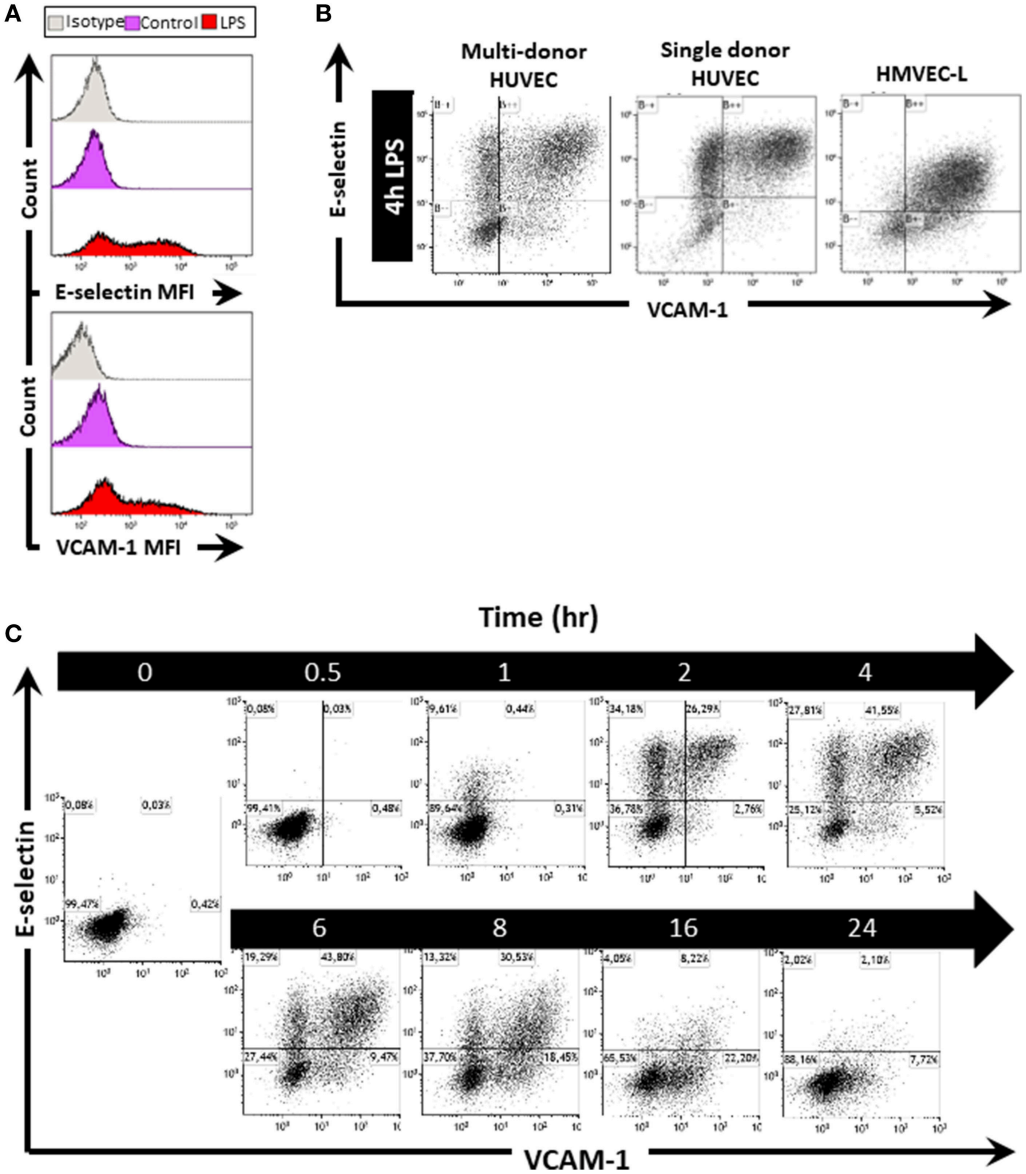
LPS stimulation of endothelial cells *in vitro* induces the formation of EC subpopulations based on E-selectin and VCAM-1 expression. **(A)** Histograms of HUVEC as one whole population show the mean fluorescent intensity (MFI) of E-selectin and VCAM-1 in untreated control and EC treated with LPS for 4 h. Isotype controls were also included. **(B)** Scatterplots of EC subpopulations based on E-selectin and VCAM-1 expression in multi-donor HUVEC, single donor HUVEC, and lung microvascular endothelium (HMVEC-L), 4 h after LPS exposure. The data shown is representative of at least four independent experiments. **(C)** Kinetics of EC subpopulation formation based on E-selectin and VCAM-1 expression in HUVEC stimulated with LPS for the indicated time periods. The data shown is representative of two independent experiments.

To investigate whether EC *in vitro* can also express E-selectin and VCAM-1 in varying patterns, we stimulated HUVEC with 1 μg/mL LPS for 4 h, after which we flow cytometrically determined E-selectin and VCAM-1 protein levels. In control cells, E-selectin and VCAM-1 expression levels were low (Figure 2A). After 4 h of LPS stimulation, the expression of both E-selectin and VCAM-1 was increased, as evidenced by the shift in the Mean Fluorescent Intensity (MFI) relative to non-stimulated controls (Figure 2A). At the cell population level, 4 h of LPS exposure surprisingly revealed the formation of EC subpopulations Figure 2B; Supplementary Figure 2A which bear similarities with the subpopulations observed in the microvasculature in the kidney. A large subpopulation of EC (approximately 50%) expressed both E-selectin and VCAM-1, while at the same time a significant subset of cells (approximately 20%) remained “quiescent” despite exposure to LPS Figure 2B; Supplementary Figure 2A. Moreover, two additional EC subpopulations were identified that expressed either E-selectin or VCAM-1 Figure 2B; Supplementary Figure 2A. Similar results were found when exposing HUVEC isolated from single donors to 4 h LPS (Figure 2B), and also human lung microvascular endothelial cells (HMVEC-L) exposed to LPS revealed the formation of these four subpopulations Figure 2B. Endothelial subpopulations identified by heterogeneous expression of E-selectin and VCAM-1 and combinations thereof after LPS exposure is therefore not restricted to HUVEC and not donor related but a common response of endothelial cells.

We next studied the kinetics of E-selectin and VCAM-1 expression in HUVEC. Upon LPS exposure, some EC started to express E-selectin as early as 1 h after start of activation by LPS, while at 2 h, VCAM-1 expression became apparent Figure 2C; Supplementary Figure 2B. Moreover, at this early timepoint, four EC subpopulations already had started to form, i.c., “quiescent” E-selectin^−^/VCAM-1^−^ (-/-), E-selectin^+^/VCAM-1^−^ (E-sel+), E-selectin^+^/VCAM-1^+^ (+/+), and E-selectin^−^/VCAM-1^+^ (VCAM-1+). These expression profiles were retained until 6 h of LPS exposure, after which the E-selectin positive subpopulation diminished in cell number, while the VCAM-1+ subset and quiescent subpopulations increased. At 24 h, most EC had returned to a quiescent state, with only a small VCAM-1+ subset (7% of all cells) still being present (Figure 2C). A quiescent endothelial cell subpopulation was constantly present at all times Figure 2C; Supplementary Figure 2B. Furthermore, these patterns of E-selectin and VCAM-1 expression were similar when exposed to different LPS concentrations, except for the lowest LPS concentration (0.1 μg/mL) which did not seem to activate the endothelial cells (Supplementary Figure 2C). Taken together, our data demonstrate that activation of endothelial cells by LPS leads to E-selectin and VCAM-1 (co)expression patterns that are dynamically changing depending on the time of exposure to LPS and which are not affected by the concentration of LPS used.

### Cell Division Is Not a Major Factor in Controlling the Ability of EC to Express E-Selectin and/or VCAM-1 When Exposed to LPS

**Figure 3:**
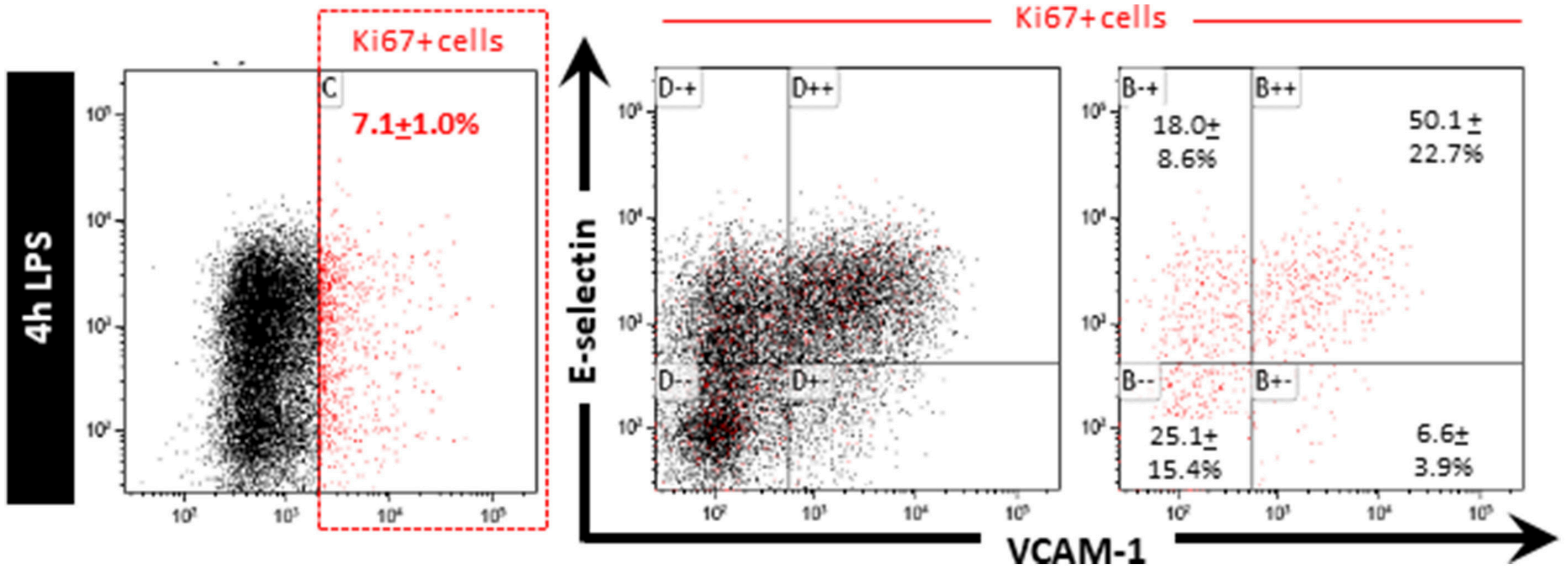
Cell division is not a major factor controlling the ability of EC to express E-selectin and/or VCAM-1 when exposed to LPS. HUVEC exposed to LPS for 4 h were stained for Ki67 after which Ki67 positive cells were gated and the extent of E-selectin and VCAM-1 expression on those cells determined. Ki67 positive cells are indicated in red. The percentage of Ki67 positive cells expressing different extents of E-selectin and VCAM-1 is reported as the mean ± SD of three independent experiments.

HeLa cells were reported to repress NF-κB activity following inflammatory stimulation while dividing as cell division appeared to have a higher cellular priority (17). Whether this also occurs in dividing endothelial cells is currently unknown. We hypothesized that the quiescent EC subpopulation was unable to express E-selectin and VCAM-1 because these cells are actively dividing. Staining for Ki67, a marker of actively dividing cells, however, revealed that the total number of Ki67 cells in the HUVEC population as a whole was only approximately 7% Figure 3. This is much less than the 25% of cells in the quiescent cell population after LPS stimulation. Moreover, when gating the Ki67 positive cells we found that they were present in all four flow cytometric quadrants Figure 3. Hence, cell division *per se* does not seem to be a major factor influencing the ability of EC to express E-selectin and/or VCAM-1 and is therefore not a likely factor contributing to the “quiescence status” of EC that do not express the adhesion molecules upon exposure to LPS.

### Endothelial Subpopulations Have Distinct mRNA Expression Levels of LPS Signaling Pathway Components and Inflammatory Genes

**Figure 4:**
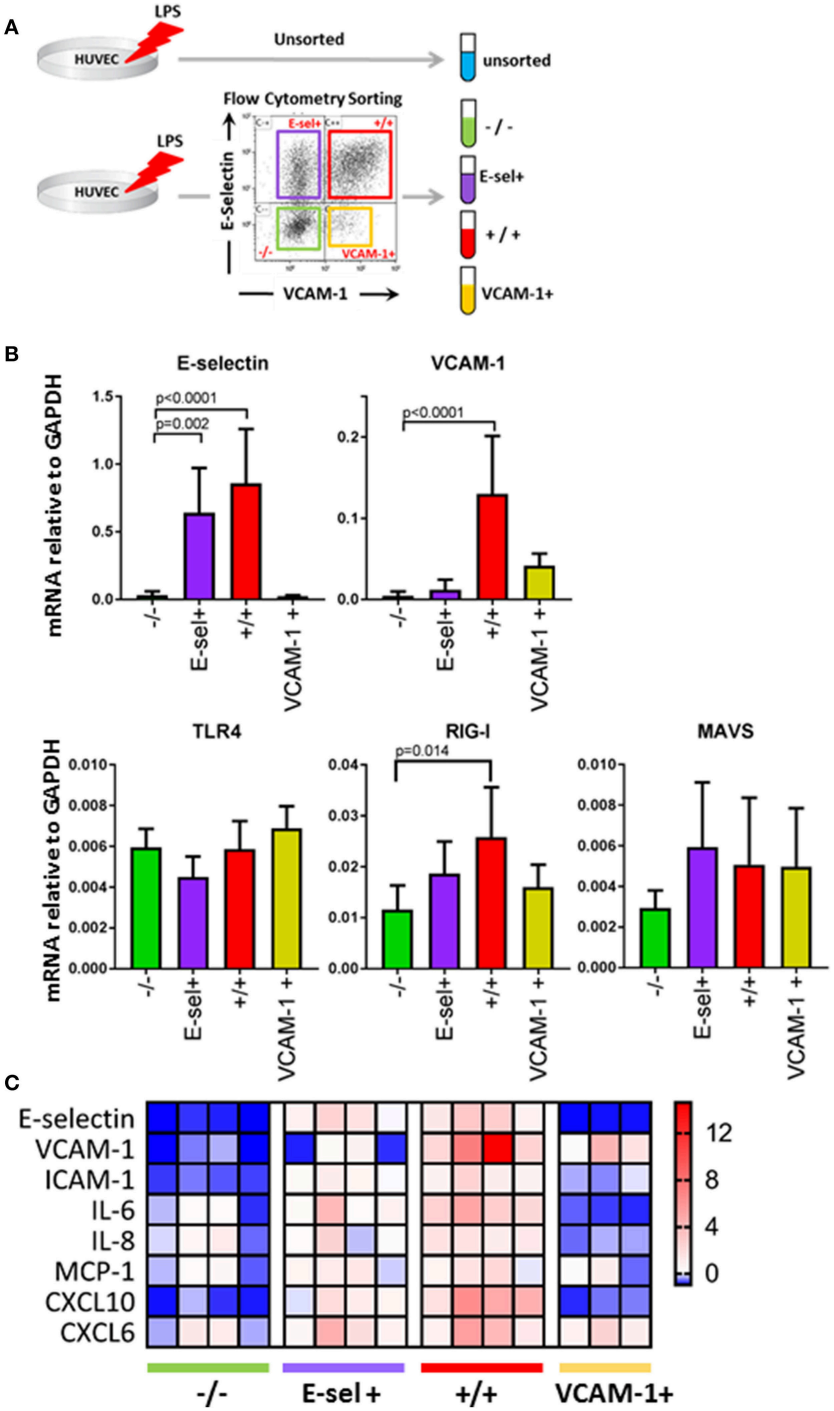
Endothelial subpopulations have distinct mRNA expression levels of LPS signaling pathway components and inflammatory genes. **(A)** Experimental design for EC sorting using FACS. **(B)** mRNA expression levels of E-selectin, VCAM-1, TLR4, RIG-I, and MAVS in sorted EC subpopulations as determined by RT-qPCR using GAPDH as the housekeeping gene. Bars represent the mean ± SD of 5 independent experiments. *p* < 0.05 is considered statistically significant. **(C)** Heatmap displaying pro-inflammatory adhesion molecule, cytokine, and chemokine mRNA levels in sorted EC subpopulations compared to unsorted LPS control EC. The data shown is from four independent experiments.

LPS induced adhesion molecule expression in EC is known to be mediated by at least two pattern recognition receptors, TLR4 and RIG-I (10, 18). We therefore hypothesized that the subpopulation distribution of EC after LPS exposure identified by E-selectin and VCAM-1 patterns was due to different subpopulations expressing LPS signaling components to different extents. To investigate this, we incubated HUVEC with LPS for 4 h and then separated the quiescent -/-, E-sel+, +/+ and VCAM-1+ subpopulations by fluorescence activated cell sorting (FACS). After sorting and confirmation of subpopulation purity (data not shown), we first determined the E-selectin and VCAM-1 mRNA expression levels Figure 4B. E-selectin and VCAM-1 mRNA levels were close to absent in the quiescent -/- subpopulation, while E-selectin mRNA was high in E-sel+ and +/+ subpopulations, and VCAM-1 mRNA was high in +/+ and VCAM-1+ subpopulations thereby corroborating the protein data. We proceeded to determine the mRNA expression levels of TLR4 and RIG-I within the different EC subpopulations. TLR4 mRNA levels were similar in all EC subpopulations including the quiescent one Figure 4B. RIG-I mRNA levels were highest in the E-sel^+^/VCAM-1^+^ cell population and expressed at a slightly lower level in the other EC subpopulations Figure 4B, while mRNA levels of MAVS, a downstream protein adaptor of RIG-I, were similar in all four EC subpopulations Figure 4B. To further investigate the inflammatory status of the four subpopulations, we analyzed the expression levels of a series of cytokines/chemokines known to be produced by endothelial cells in inflammatory conditions. While both the quiescent and the VCAM-1+ subpopulations expressed the lowest levels of these genes, the +/+ subpopulation exerted the most pronounced inflammatory phenotype Figure 4C. These results demonstrate that upon LPS exposure, there are distinct endothelial subpopulations with varying LPS signaling components and inflammatory phenotypes within a HUVEC cell population.

### TLR4 and RIG-I Differentially Regulate LPS-Mediated E-Selectin/VCAM-1 Expressing Subpopulations

**Figure 5:**
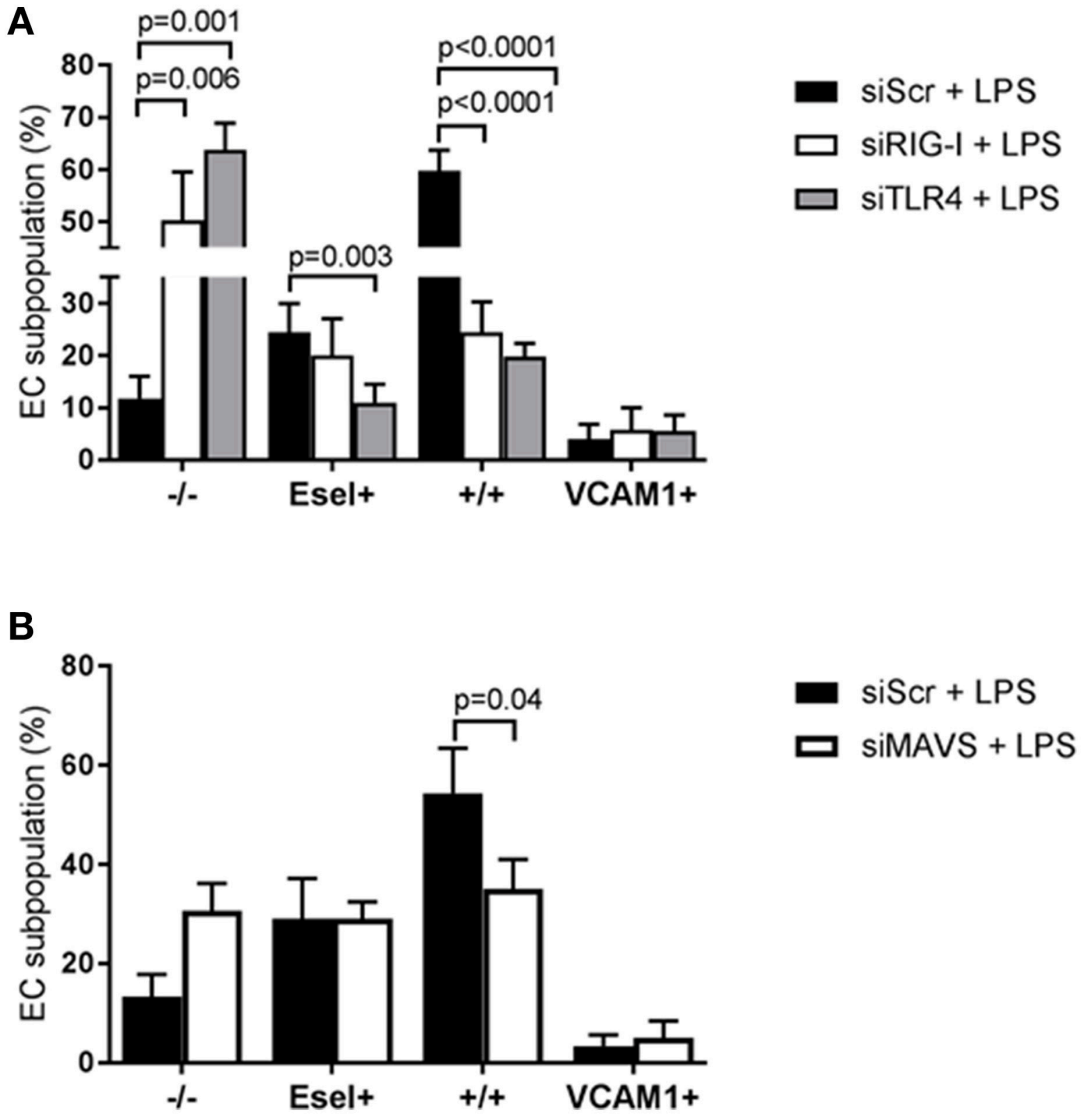
TLR4 and RIG-I-MAVS differentially regulate LPS-mediated E-selectin/VCAM-1 subpopulation formation. **(A)** The effect of small interfering RNA (siRNA)-based RIG-I and TLR4 knockdown on the formation of LPS-induced EC subpopulations based on E-selectin and VCAM-1 expression after 4 h of LPS exposure compared to scramble siRNA (siScr) controls. Bars represent the mean ± SD of 4 independent experiments. **(B)** The consequence of MAVS knockdown on the formation of the EC subpopulations. Bars represent the mean ± SD of 3 independent experiments. *p* < 0.05 is considered statistically significant.

Since both receptors are expressed in all EC subpopulations, we proceeded to examine the effect of TLR4 and RIG-I siRNA knockdown on the formation of E-selectin and VCAM-1 expressing subpopulations in HUVEC upon LPS exposure. Knockdown of both TLR4 and RIG-I resulted in diminished protein levels of both E-selectin and VCAM-1 when analyzing the HUVEC population as a whole (data not shown) corroborating previous findings (10). While only TLR4 knockdown resulted in inhibition of the E-sel+ subpopulation formation, knockdown of either TLR4 or RIG-I resulted in a major inhibition of +/+ subpopulation formation Figure 5A. As a consequence, the -/- “quiescent” subpopulation increased Figure 5A. In contrast to the inhibitory effect observed on the E-sel+ or +/+ subpopulations, knockdown of TLR4 or RIG-I did not inhibit the VCAM-1+ subpopulation from forming Figure 5A. Similar to the effects of RIG-I knockdown, MAVS knockdown inhibited the formation of the +/+ subpopulation with no effect on E-sel+ or VCAM-1+ subpopulation formation Figure 5B. These results suggest that TLR4 predominantly controls the formation of E-sel+ cells and that both TLR4 and RIG-I control the formation of the E-sel+/VCAM-1+ population.

### LPS-Mediated E-Selectin/VCAM-1 Expressing HUVEC Subpopulations Are Regulated by Different Downstream Signaling Mechanisms

We previously found that the absence of RIG-I or MAVS inhibited nuclear translocation of the NF-κB p65 subunit (10). Moreover, the NF-κB p65 subunit translocated from the cytoplasm to the nucleus in only 26% of EC 30 min after LPS stimulation (Supplementary Figure 3). Apart from NF-κB signaling, activation of p38 MAPK signaling in LPS exposed endothelial cells has previously been reported (19, 20). We therefore investigated whether one or both of these two signaling pathways were affecting the formation of one or more subpopulations employing pharmacological tools and if so, to what extent the subpopulations were affected.

**Figure 6:**
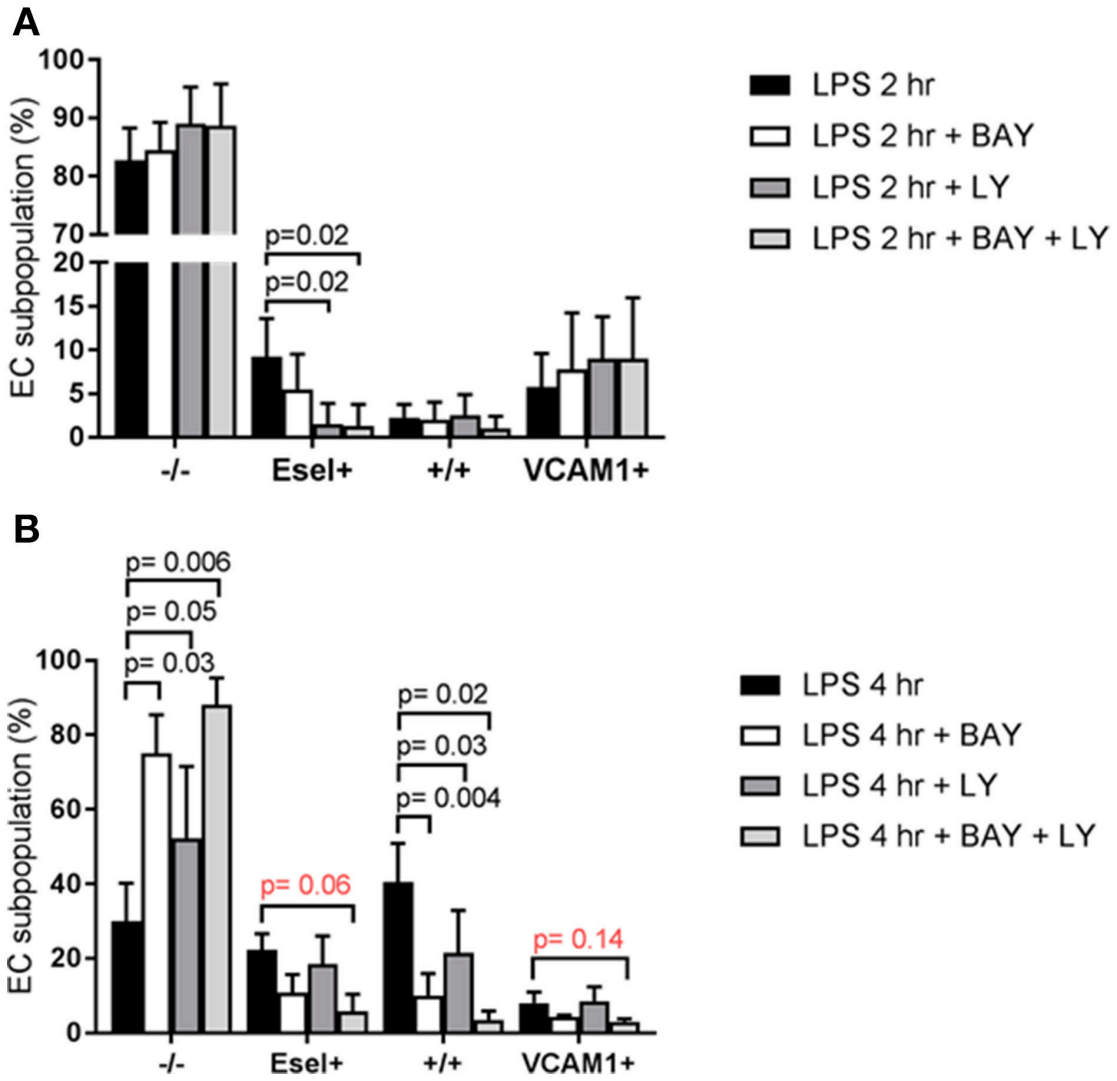
LPS-mediated E-selectin/VCAM-1 HUVEC subpopulations are regulated by different downstream signaling mechanisms. **(A)** The effect of pharmacologically inhibiting NF-κB with BAY 11-7082 (BAY) and p38 MAPK with LY2228820 (LY) pre-treatment on the formation of the LPS-induced EC subpopulations based on E-selectin and VCAM-1 expression, after 2 h of LPS exposure; and **(B)** after 4 h of LPS exposure. Bars represent the mean ± SD of 4 independent experiments. *p* < 0.05 is considered statistically significant.

When examining the subpopulation distribution after 2 h exposure to LPS we found that p38 MAPK inhibition using LY exerted a major inhibitory effect on the formation of the E-sel+ subpopulation Figure 6A. In contrast, NF-κB inhibition using BAY did not significantly influence the formation of any of the subpopulations at this early time point. At 4 h LPS exposure however, blockade of NF-κB and p38 MAPK had inhibited the formation of the +/+ subpopulation (Figure 6B). In addition, the combination of NF-κB and p38 MAPK inhibition strongly diminished the formation of the E-selectin+/VCAM-1+ expressing subpopulation Figure 6B. Formation of the VCAM-1+ subpopulation was not affected by inhibition of NF-κB and/or p38 MAPK. Also, inhibition of both NF-κB and p38 MAPK did not fully block the formation of the subpopulations, suggesting that other signaling pathways contribute to their formation.

## Discussion

Sepsis is a life-threatening condition which is characterized by progressive host dysregulation, following a known or suspected infection (1). Sepsis pathophysiology is still not fully understood which has hampered the development of therapeutic options counteracting organ failure in patients with sepsis. Due to their location, endothelial cells are among the first cells to respond to systemic pathogens or bacterial products (3). A hallmark of sepsis-related organ dysfunction is (micro)vascular dysfunction which include endothelial activation. This results in immune cell infiltration which can be detrimental for organ function (5). The current study aimed to explore the molecular control of early LPS mediated endothelial activation in relation to previously reported heterogeneous EC responses found *in vivo*. Our findings reveal that in the kidneys of mice challenged with LPS, EC between different microvascular compartments and also within the same microvascular bed, exert a heterogeneous phenotype as identified by different patterns of E-selectin and VCAM-1 protein expression. Moreover, we observed that *in vitro* in endothelial cell cultures, subpopulations appear shortly after the start of LPS exposure that exert a phenotypic E-selectin/VCAM-1 heterogeneity similar to that observed in the mouse kidney. These endothelial subpopulations have distinct inflammatory phenotypes, with the E-selectin/VCAM-1 double positive subset showing the highest level of activation. The formation of the subpopulations is differentially regulated by distinct signaling mechanisms, both at the level of TLR4 and RIG-I, and at the level of the NF-κB and p38 MAPK pathways. Strikingly, we found a quiescent population of cells that was not only devoid of E-selectin and VCAM-1 expression but was also lacking the expression of other proinflammatory cytokines and chemokines despite exposure to LPS.

It is well known that EC acquire organ- and tissue-specific identities to support the unique requirements of various organs in the body (21). The differential expression of E-selectin and VCAM-1 in different renal microvascular compartments of LPS-treated mice reported in this study corroborates previous observations from our group (6, 7). In addition, we revealed that within the same microvascular compartment, endothelial cells heterogenically express E-selectin and VCAM-1 following LPS challenge. While phenotypic differences between adjacent EC conditioned in the same environment were previously shown for endothelial barrier antigen in capillaries of the brain of rats (22) and for Tie-2 in mouse xenografts associated tumor neovessels (23), heterogeneous expression of cell adhesion molecules within individual microvascular segments has to our knowledge not been reported before. Interestingly, endothelial cells *in vitro* exhibited a similar phenotypic heterogeneity in E-selectin and VCAM-1 (co)expression patterns. The four subpopulations of EC, each with a different composition of E-selectin / VCAM-1 (co)expression, did not only appear in multi-donor HUVEC, but also in single-donor HUVEC and in human lung microvascular endothelial cells (HMVEC-L) exposed to LPS. From this we conclude that the observed heterogenous responsiveness is not a specific attribute of HUVEC but rather a common response of endothelial cells exposed to LPS. Of notice, HMVEC-L displayed a different composition of EC subpopulations, which could be attributed to a specific lung-microvascular phenotypical characteristic to support dedicated biological functions in the lungs. The E-sel^+^/VCAM-1^+^ subpopulation also expressed the highest levels of inflammatory mediators IL-6, IL-8, MCP-1, CXCL6, and CXCL10, implying that this subpopulation has a more generalized higher activation status than the other three subpopulations. This finding is in line with a previous study that showed that high IL-8-secreting EC produced higher levels of endothelial adhesion molecules, chemokines, and cytokines compared to their low IL-8-secreting EC counterparts (24). Why CXCL6, a chemoattractant for neutrophils, was specifically expressed in the VCAM-1+ subpopulation remains to be clarified. Follow up studies will determine the full nature and extent of activation of the cells in these subpopulations.

Intriguingly, we found a subpopulation of EC that did not express E-selectin or VCAM-1 despite prolonged exposure to LPS. Gene expression analysis of this sorted subpopulation of cells showed that these cells had a broader “quiescent” phenotype since they also did not express inflammatory cytokines and chemokines upon LPS exposure. One possible explanation for these findings could have been that these cells lack LPS-signaling machinery, but we showed that that was not the case as this population was expressing both TLR4 and RIG-I, two of the main LPS-signaling molecules in endothelial cells. Another explanation for these findings might have been that the cells in the “quiescent” phenotype subpopulation were dividing and thereby subjected to repression of NF-κB–driven inflammation as was previously reported in HeLa cells (17). However, we found only 7% of the total HUVEC population to be proliferating, while the quiescent population represented around 20% of cells. Moreover, Ki67 positive, proliferating cells were also able to express E-selectin, which confirmed previous observations (25, 26) and VCAM-1. Hence, cell proliferation does not appear to be a major contributing factor leading to the “quiescent” status of these cells. Since exaggerated and prolonged inflammatory responses can be detrimental to cells, a negative feedback loop mechanism exists that regulates the magnitude and duration of inflammation (27). An example of such is the activation of the zinc finger protein A20 which limits inflammation downstream of the NF-κB pathway. A20 mRNA levels were, however, comparable in all EC subpopulations (data not shown) including the quiescent EC subpopulation which suggests that an A20-dependent inhibitory feedback mechanism is not controlling the quiescent status of these E-selectin^−^/ VCAM-1^−^ cells. Why these cells remain quiescent despite LPS exposure is currently unknown. We will investigate this further since understanding the molecular mechanisms associated with this quiescent phenotype may eventually be exploited for therapeutic strategies to inhibit endothelial activation in the setting of sepsis.

**Figure 7:**
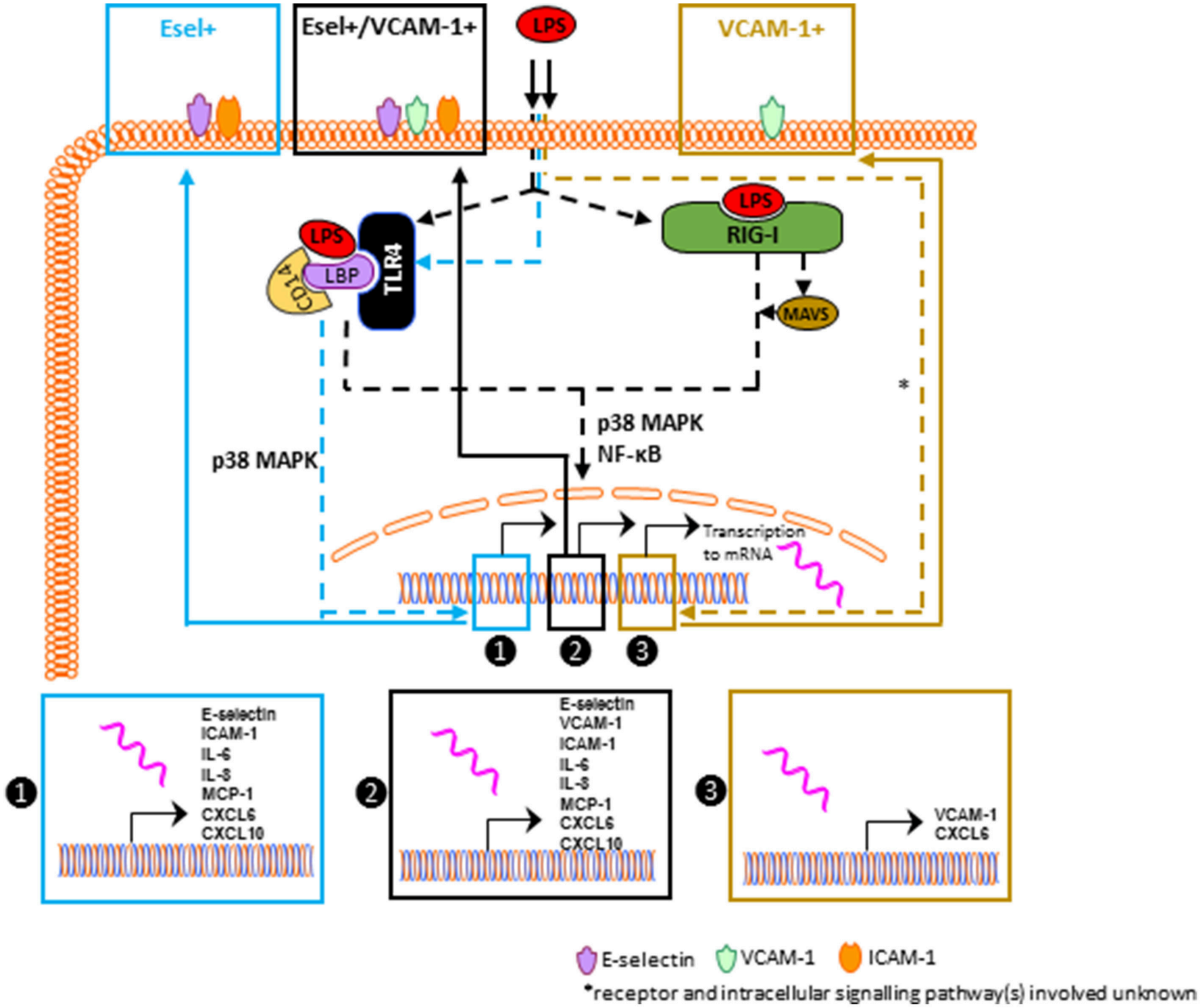
Proposed cellular regulatory mechanisms that control the formation of LPS-mediated EC subpopulations. Dashed lines represent pathways involved in transcriptional control of pro-inflammatory molecules, and solid lines represent pro-inflammatory proteins on EC. The blue, black, and brown lines represent intracellular signaling pathways that determine E-sel+, E-sel+/VCAM-1+, and VCAM-1+ subpopulation formation.

We explored the possibility that the heterogeneous responses by the EC subpopulations were attributed to intrinsic cellular differences in the functionality of the LPS signaling components. Using siRNA-based knock down we investigated the role of TLR4 and RIG-I in this process. While TLR4 mediated both E-sel+ and E-sel^+^/VCAM-1^+^ subpopulation formation, RIG-I mainly had a role in E-sel^+^/VCAM-1^+^ subpopulation formation. Knocking down MAVS, a RIG-I adaptor protein, did not affect the formation of E-sel+ and VCAM-1+ subpopulations. Pharmacological inhibition studies showed that NF-κB and p38 MAPK were the predominant controlling pathways for the formation of the E-sel+ and E-sel^+^/VCAM-1^+^ subpopulations (see Figure 7). The fact that the VCAM-1+ population at early time points of LPS stimulation was not affected by any of the pathways investigated raises the question how this subpopulation is being formed when endothelial cells are exposed to LPS. One way to establish a clear role of LPS signaling in VCAM-1+ subpopulation formation is by performing knockdown experiments and examining the activation status when the VCAM-1+ subpopulation is at its peak (16 h after LPS stimulation, Supplementary Figure 2B). At 16 h of LPS exposure, it is highly likely that secondary or tertiary responses have become part of the equation following the release of pro-inflammatory cytokines, such as IL-6 and IL-8. Exposing naïve HUVEC to cytokines (e.g., IL-6) has been shown to increase E-selectin, VCAM-1, and ICAM-1 (28). Considering the pharmacological importance of the findings reported here, we will investigate this issue in more detail in HUVEC subpopulations using kinase activity platform technology.

In conclusion, renal endothelial cells exert a heterogeneous pattern of E-selectin and VCAM-1 expression following LPS challenge, in different as well as within the same microvascular segments. Such a heterogeneous response was recapitulated *in vitro* when HUVEC and human lung microvascular endothelial cells were exposed to LPS. The here identified and described endothelial subpopulations have distinct inflammatory phenotypes and are regulated by different signaling mechanisms. At this point, it is not known whether signaling differences contributing to the heterogeneous expression of E-selectin and VCAM-1 has biological relevance, nor what the pharmacological ramifications are. Such biological relevance should be investigated in *in vivo* settings, as the molecular signatures of endothelial cells differ depending on their specialized functions and microenvironment both in homeostasis and during inflammatory responses (21, 29). Follow up studies will aim to further investigate the underlying molecular pathways in *in vitro* and *in vivo* as a lead for future therapy choices to attenuate activation of microvascular endothelial cells in the organs of critically ill patients with sepsis.

## Ethics Statement

This study was carried out in accordance with the recommendations of the animal ethics committee of the University of Groningen.

## Author Contributions

JM and GM shared the conceptualization. JM, GM, and ED designed the study. ED, JP, BE, and JM performed the experiments and analyzed the data. MM provided valuable input on the statistical analysis. ED, JM, and GM wrote and edited the manuscript. All authors critically revised the manuscript and approved the submitted version.

### Conflict of Interest Statement

The authors declare that the research was conducted in the absence of any commercial or financial relationships that could be construed as a potential conflict of interest.
