# Supplementary material for: Identification of LPS-Activated Endothelial Subpopulations With Distinct Inflammatory Phenotypes and Regulatory Signaling Mechanisms
Source: Front Immunol. 2019 May 24;10:1169. doi: 10.3389/fimmu.2019.01169 (PMC6543489; doi:10.3389/fimmu.2019.01169)
Supplement: Supplementary file 1 [file Data_Sheet_1.PDF]

A

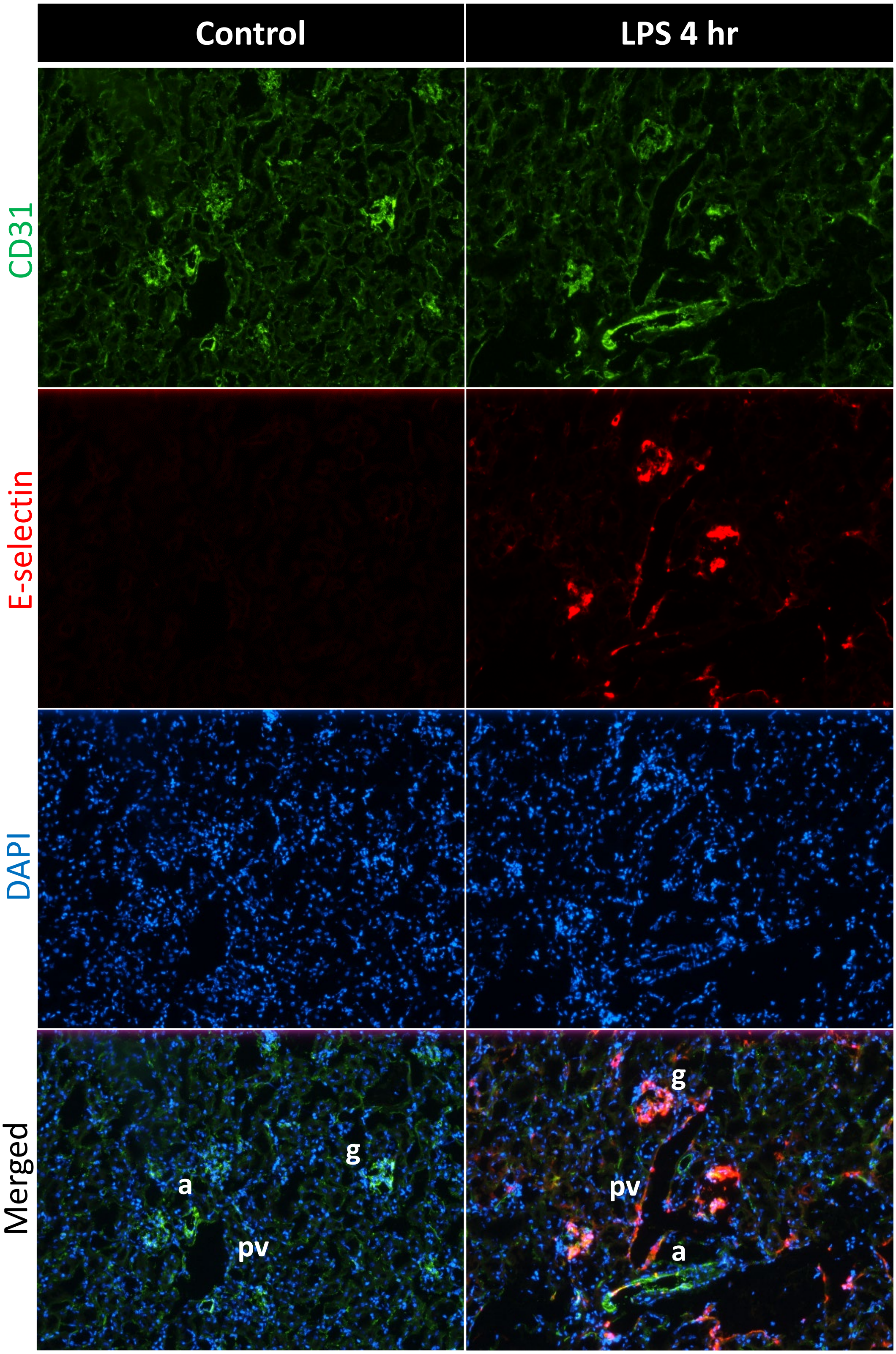

Supplemental Figure 1(A). Colocalization of CD31 and E-selectin expression in mouse kidney microvascular compartments. The microvascular compartments of mouse kidney were annotated as 'a' (arterioles), 'g' (glomerulus) and 'pv' (post-capillary venules). The images were captured at 100x magnification with equal exposure times.

B

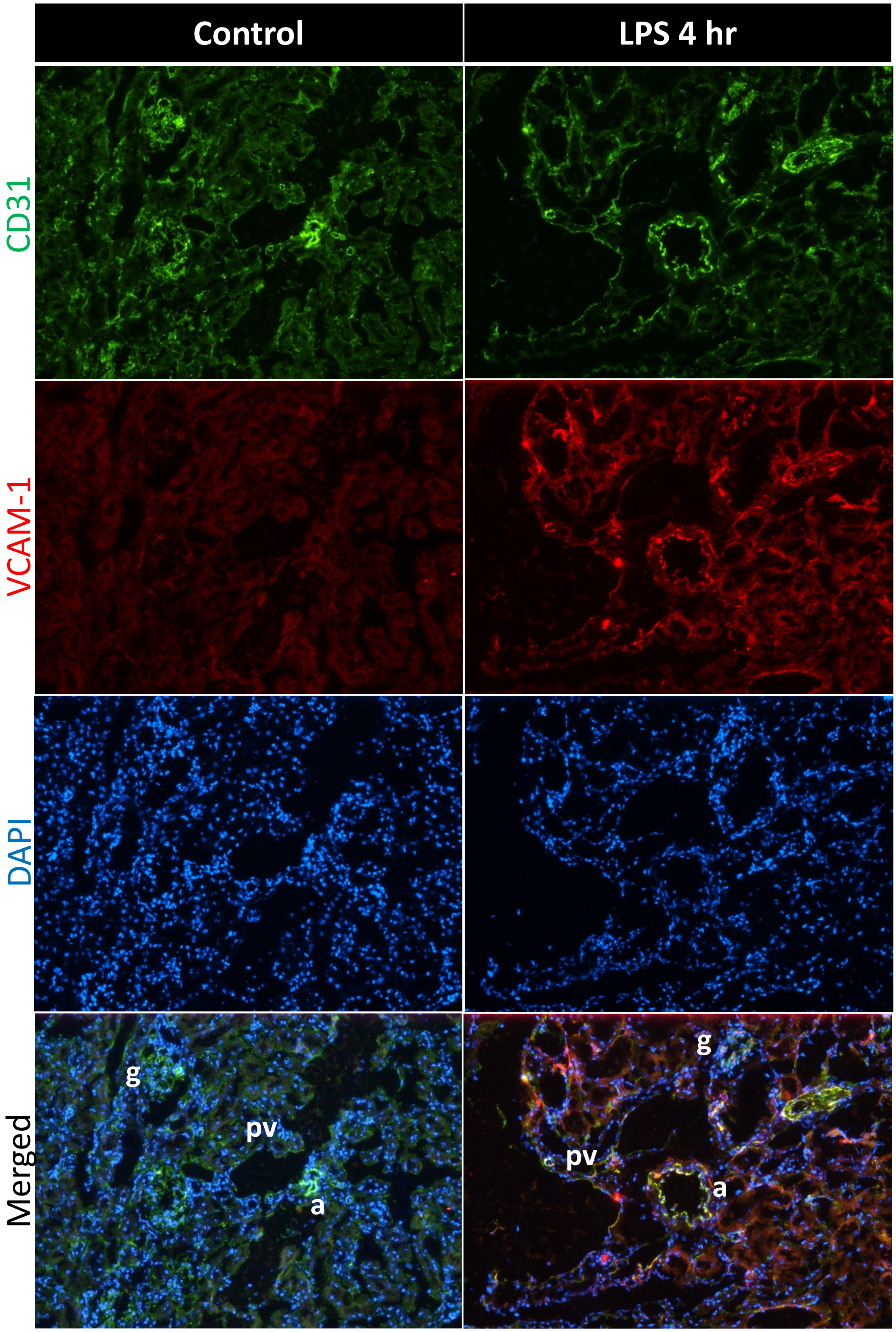

Supplemental Figure 1(B). Colocalization of CD31 and VCAM-1 expression in mouse kidney microvascular compartments. The microvascular compartments of mouse kidney were annotated as 'a' (arterioles), 'g' (glomerulus) and 'pv' (post-capillary venules). The images were captured at 100x magnification with equal exposure times.

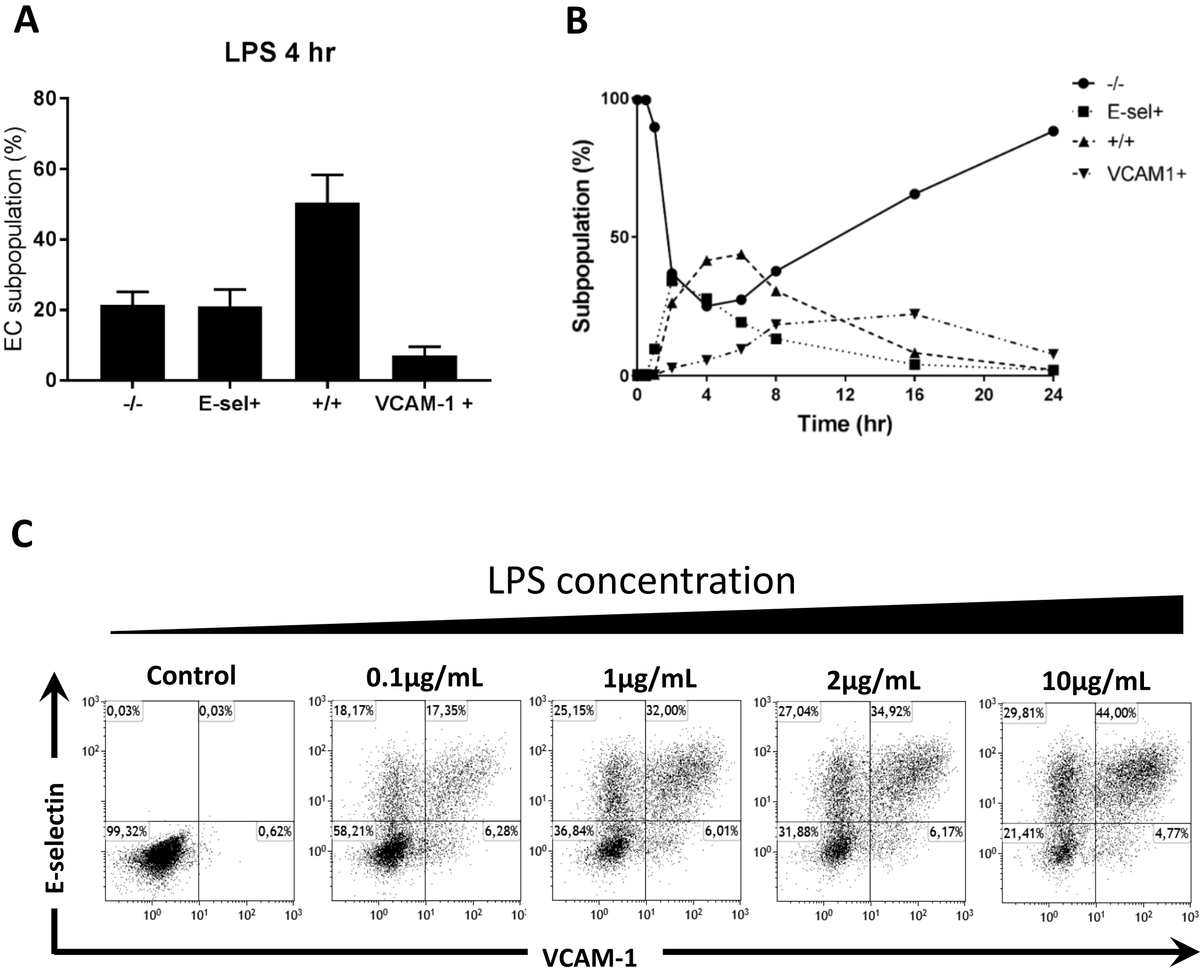

Supplemental Figure 2. LPS induced endothelial subset formation based on E-selectin and VCAM-1 expression is concentration-independent. (a) EC subpopulations formed based on E-selectin and VCAM-1 expression following exposure with LPS for 4 hr, as determined by flow cytometric analysis. Bars represent average + SD of four independent experiments. **(b)** Kinetics of EC subpopulation formation based on E-selectin and VCAM-1 expression following LPS stimulation for indicated times. The data is representative of two independent experiments. **(c)** Flow cytometric scatterplots representing EC subpopulations formed based on E-selectin and VCAM-1 expression following LPS stimulation with indicated concentrations. The data represent three independent experiments.

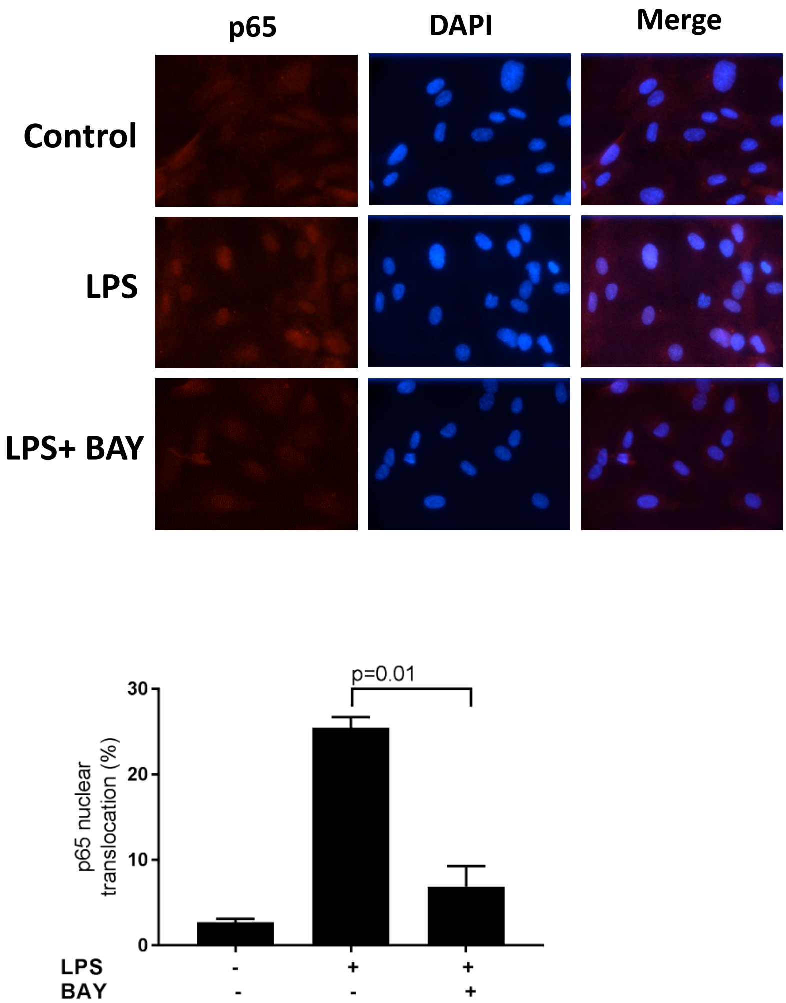

Supplemental Figure 3. BAY 11-7082 blocked p65 nuclear translocation in LPS-stimulated HUVEC. LPS-stimulated HUVEC and non-stimulated controls were stained with rabbit anti-human p65 antibody (Cat. No. #D14E12, Cell Signaling Technology, Danvers, MA, USA) diluted in washing buffer (PBS containing 0.5% (w/v) BSA and 0.05% (v/v]) Tween 20 (Sigma, St. Louis, Missouri, USA)) for 1 hr. HUVEC were then incubated with Alexa Fluor®555-conjugated donkey anti-rabbit secondary antibody (A-31572, Life Technologies, The Netherlands) for 45 min. The cells were mounted in Aqua/Polymount medium containing DAPI (1.5 µg/mL, Polysciences, Warrington, PA, USA). Fluorescence images were taken with equal exposure times using appropriate filters with a Leica DM4000B fluorescence microscope equipped with a Leica DFC345FX digital camera (Leica Microsystems Ltd., Germany) and Leica LAS V4.5 Image Software. Nuclear translocation of p65 was quantitated in 250 cells. Bars represent the means of three independent experiments + s.d..
